# Nasal Catarrh

**Published:** 1877-08

**Authors:** J. C. LeHardy

**Affiliations:** Savannah, Ga.


ATLANTA

MEDICAL AND SURGICAL JOURNAL

VOL. XV.] AUGUST—1877. [NO. 5

### Original Communications.

### NASAL CATARRH.

BY J. C. LEHARDY, M.D., SAVANNAH, GA.

There are certain diseases which, like most exotic plants, may be imported where they were before unknown, and like them also, provided they find a suitable soil and climate, will thrive and be reproduced for an indifinite length of time. Whether the seed, the cell, the plant or "what not," producing these diseases be brought through the channels of commerce, or transported by "ill winds," it is here needless to discuss, as in either case it would be impossible to prevent the *importation.* Our aim is to combat the disease and destroy the germ. These reflections were suggested by the appearance in our town, some ten years ago, of a troublesome affection of the mucous membrne of the naso-pharyngéal passages, or which is more commonly called catarrh of the nose. As a great number of cases originated, then, in the commercial part of the city, and as at the same date, guano was carried constantly through the streets and along the wharves, the *causa latens* was placed to the credit of this article, and the city authorities ordered the hauling and storing of guano to be restricted to certain limits. The disease, however, continued to spread, affecting persons of all ages and stations "without regard to color or previous condition."

*The mode of invasion* is very insidious and often gives so little annoyance as to be overlooked for a long time. In some instances there is a mucous secretion which is believed to be produced by a "cold," in others a burning and itching sensation replaces the discharge of mucous and is at times annoying, inducing the picking at the nose with the finger nail.

Scabs now begin to form, and their presence in the nose becomes a constant source of annoyance as they require to be blown out in the handkerchief. These scabs at first transperent, become yellowish, and finally of a greenish gray or black color, when they emit a nauseous odor. However, the person affected rarely perceives this odor himself, on account of a partial loss of the sense of smell, resulting from the spreading inflammation of the schneiderian membranes. The first intimation received by the sufferer is generally from some member of the family or a friend who discovers a peculiarly nauseous, almost cadaveric smell issuing from the nose or from the mouth, whilst speaking; now, for the first time a physician is consulted and the astonished patient learns that his disease is of months, perhaps of years standing.

*The physical appearance* of the disease, which is best discovered in the sun-light either direct or reflected, is as follows: The mucous membrane covering the nasal passages as far as it can be seen presents a deep red color, the result of a chronic inflammation, and in many cases it is so much thickened as to resemble polypoid growths, almost closing the entire passage. If the throat be now examined by direct light on depressing the tongue, the upper portion of the pharynx will be found inflamed, thickened and generally covered with a muco-purulent secretion. In using the reflecting mirrors this inflammation will be seen to extend upwards, involving the openings of eustachian tubes and the posterior nares. In the more advanced cases, on the floor and septum will be seen a dark scab, which, being taken hold of by broad-bladed forceps, can be removed entire; its forcible removal is however usually followed by bleeding from portions of the surface to which it was attached. This scab varies in size and thickness, is tenacious, and of a grayish green color, emitting a very disagreeable smell.

*The miscroscopic examination* of this scab, as might be expected from its position and slow mode of formation, will bring before the astonished eye of the looker on such a multifarious array of objects as would set the mind of an aspiring student wild in the field of *discovery*. The animal, vegetable and mineral creation are all represented here—from the dust of age to king cotton; bright crystals, rich woolen tissues—the cryptogams, the algæ, as well as the more complex vegetable tissues have their place in the panorama passing before the eye as the slide is slowly moved. However, after repeated examinations, it will become apparent that they are foreign substances, caught in the nasal secretions in the act of breathing.

In every scab of a well marked case of naso-pharyngeal catarrh there may always be found a very large amount of mucus cells, also exudation cells, puss cells and granules, large and small epithelial scales and occasionally blood cells and fibrine filaments; intermixed with these, in almost every specimen examined, will be found a greenish coloring substance which appears amorphous, and floats freely among the granules, or seems incorporated with the epithelial scales. Then again, in the more liquid preparations, there are sometimes perceptible small rounded cells, either single, in cluster, or in lines, floating rapidly on the surface—these I believe to be the very "*bacteriæ*," or a very closely allied species of the *renowned disease generator.*

I have not, however, so far as my researches have gone, been able to trace the cause of this disease to the presence of that remarkable ferment, for the reason that in the examinations made of the fresh mucous secretions collected in site in the various stages of the disease, I have failed to find them except in three instances, and in these, they were very few in number, single cells, and floating. In the green amorphous substance so constantly found in this complaint, there may be found a cause for this disease—with my means of examination I cannot point to it with certainty, as it presents no organization.

*The Pathology.*—It sometimes occurs that, while examining a patient for some other ailment, a recently developed case of this nasal catarrh comes to the physician's notice. In such a case, a small portion only of the mucous membrane lining the septum in one or both nostrils may be found inflamed and of a bright red color, it is usually covered with a perfectly limpid secretion, which, on being wiped away, discovers, on examination with a lens of three to five diameters, small points of erosion dotted over its surface.

In examing the same patient a few months later, the inflammation is seen to have extended along the septum toward the bones, on the floor of the lower meatus, and on the mucous membrane covering the upper and lower external cartilages of the alæ. So far, the sense of smell is not in the least impaired. Months after this, on again examining the same individual, the whole of the mucous membrane covering the turbinated bones, and lining the different cells connected with the nose, may be found involved in the disease. The color of the affected parts, at first bright, has turned to a deeper red; the erosion has extended over a large surface; the sense of smell has diminished in proportion to the extent of inflammation involving the mucous membrane covering the nasal fossæ and septum, and where the olfactory nerve is distributed. An examination of the pharynx at this stage indicates that the disease has spread over its whole upper surface, the mucous membrane is thickened, eroded and covered with a muco-purulent discharge.

Scabs are formed as the disease progresses; first on the septum, then on the floor of the lower chamber, then higher up on the turbinated bones. These scabs, at first thin and transparent, become grayish yellow, and finally dark grayish green. Pains are felt in the frontal cells, a thick muco-purulent discharge is constantly hawked up from the pharynx, and a general feeling of discomfort about the head is complained of.

The general health becomes affected, the digestion sluggish, the blood impoverished, the skin colorless or of a leaden hue, and great depression of spirits follows, principally amongst women, who feeling that they are loathsome, shrink from company. This condition of things may last for years without any loss of cartilage or even ulceration of the mucous membranes. There are cases where ulceration takes place in the soft tissues at the base of the cartilage of the septum; this, however is always occasioned by the pressure and irritation of the finger nail in endeavoring to remove scabs constantly forming higher up in the nasal passages. The mucous membrane and sub-mucous areolar tissues are then the only parts involved in this affection.

It will hardly be necessarry to occupy the time of the reader with a *diagnosis* of this disease, as its slow progress and slower decline distinguish it at once from *influenza* or *coryza*. From the appearance at a certain stage it might easily be mistaken for syphilitic *ozœna*, but a careful investigation into the origin and progress of the disease will unmistakably mark the difference. Should the distinction not be recognized, however. at this period, its syphitic origin is sure to declare itself the moment that the bony tissue becomes involved.

The prognosis is always favorable, but in order to succeed in securing the time necessary to secure the disease, the physician should always prepare his patient for a long siege, otherwise he will become weary and dissatisfied, will abandon a regular treatment and throw himself headlong into the realms of *patent medicines*, or into the hands of all manner of quacks and itinerant doctors, and, at last, failing in his *search for a cure*, will become a confirmed melancholic.

*Treatment.*—Simple as the disease appears, limited, as it is, to a very small region of the body, and superficial as it remains during its whole course, it has baffled all efforts at speedy cure, whenever allowed to penetrate deeply into the complex and wonderful recesses of the nose and its appendages.

Ten years of constant experience in the treatment of this disease, has failed to bring forth an antidote—a specific cure. In that time, I have, however, succeeded in simplifying the treatment, and in shortening considerably the time of its duration.

Success rests principally in the restoration of the functions of the body to a healthy standard; in giving to the blood the fibrine and red corpuscles that have been diminished through the effects of the disease; in removing the stench from the nostrils of the affected one, which has naturally been a barrier between himself and social life, depriving him of its cheerful and healthful influence. Finally a cure is the reward of the combined efforts of physician and patient.

When the patient is anæmic, with impaired digestion, his liver torpid, I have found a combination of small doses of mercury with iron and quinine to answer very well in restoring the secretions and imparting renewed vigor.

℞.—Hydr. chl. corros. - - - - gr. i@ij

Tinct. ferri. chlor. - - - - - ℥j

Elixir cort. calisayæ (detannized) - - ℥v

M.

Dose—A teaspoonful in a wineglassful of water three times a day at meals.

At the same time his diet should be restricted, and should consist of meats and meat juice, milk, eggs, and bread; that is, to be light, nourishing and blood-making. With the meat and eggs some preparation of pepsin should be given, until powers of digestion are restored. Out-door exercise should be strictly enforced.

In those cases where the functions of the liver are natural, but where the nervous system is enfeebled, the following combination generally suffices to restore the tone in the nervous element:

℞.—Acid. phosph. dil. - - - - - ℥j
Ferri. pyrophosph. - - - - grs. c
Sulphatis strychniæ, - - - - grs. ij
Elixir gentian vel cort. calisaya detannized, ℥v
M.

Dose—A teaspoonful three times a day, in a wineglass of water.

When anæmia is the most predominant symptom, in addition to one of the foregoing prescriptions, I give, for a time, a pill containing:

℞.—Sulph. ferri exsic. - - - - grs. xxx
Sulph. manganes, - - - - grs. xxx
Sulph. quiniæ, - - - - grs. xlv
Extract gentian, - - - - - q. s.

To be made into thirty pills. Take one three times a day, at meals.

The bowels should be kept regular and the patient's body and mind engaged in some active or recreating work.

*The Local Treatment* is always unpleasant, and generally painful, and for this reason I have simplified it as much as I possibly could. In the first place, the patient should be well instructed how to use an injection, or douche, through the nostrils. (I prefer the syringe, for the reason that the force of the current can always be regulated by the patient, himself.) When he can open his mouth sufficiently and breathe whilst the current is passing from one nostril to the other, and he made to understand that all the water must be allowed to flow out of the nasal passages before breathing through them, then he can be intrusted with that part of the treatment. During a ten years constant application of this method, I have not met with a case of inflammation of the middle ear produced by an injection, when used as above directed.

A solution of common salt, in warm water, has given better results than any other injection I have used; it is generally soothing, and washes out the passages very well. When this application is made three times a day, and the passages are well cleansed, no hard scab has time to form, the nauseous smell soon disappears, and the disease is checked in its progress.

When the disease is on the wane, light astringent injections may occasionally be used to an advantage. Acetate of lead, tannic acid, sulphate of quinine, may be used in weak solutions.

The patient should also be instructed how to mop the upper part of the pharynx, by a tongue depressor, if necessary, and to make local applications there with a curved camel's hair brush, or mop made with cotton wool; for such applications, I prefer a solution of chlorate of potash or of common salt, where there exists much irritation; and of turpentine when the circulation is sluggish and the parts are covered with a muco-purulent secretion.

Having the patient conversant with the use of the mop, the nasal passages cleansed and the pharynx attended to daily; the moment that these means are found to have caused a check in the progress of the disease, is the most favorable time for the physician to begin the local curative applications.

Of these I have found nitrate of silver to answer best, and use it almost exclusively. When a strong impression requires to be made on the mucous membrane, I use a solution of one hundred to one hundred and twenty grains to the ounce, with a very fine atomizer, washing off the parts immediately after with a solution of salt. When a stimulating influence is desired, a solution of ten to forty grains is best. Such applications should be made over the nasal passages and the pharynx once every fourth, sixth, or seventh day—judging from the effects produced—until the disease is entirely cured; otherwise, you will have the mortification of going back over the same routine with your patient, or of seeing him leave you dissatified.

The result of the above treatment will generally be as follows: The appetite returns, the coloring of the skin improves gradually, the weight of the body increases, the spirits become more buoyant, and your patient is not only grateful for the benefits obtained, but is anxious, among women, especially, to carry on strictly the directions of the physician until an entire restoration to health is obtained. Men generally abandon the treatment before its completion, and the result is, that, while there are some who do recover, many have a return of the disease and all of its unpleasant symptoms.

